# Se@SiO_2_@mSiO_2_ nanoparticle inhibits cellular oxidative stress and Wnt/β-catenin pathway by inhibiting AGGF1 expression in the treatment of prostate cancer

**DOI:** 10.1515/jtim-2026-0076

**Published:** 2026-10-23

**Authors:** Jingcheng Lyu, Mingyu Chang, Lianhui Sun, Ruiyu Yue, Xijian Liu, Guangjian Fan, Aihong Chen, Wei Huang, Ye Tian, Boyu Yang

**Affiliations:** Department of Urology, Capital Medical University Beijing Friendship Hospital, Beijing, China; Institute of Urology, Beijing Municipal Health Commission, Beijing, China; Department of Urology, Beijing Electric Power Hospital of State Grid Corporation of China, Beijing, China; Department of Immunology, School of Cell and Gene Therapy, Songjiang Research Institute, Shanghai Jiaotong University School of Medicine Affiliated Songjiang Hospital, Shanghai, China; College of Chemistry and Chemical Engineering, Shanghai University of Engineering Science, Shanghai, China; Precision Research Center for Refractory Diseases, Institute for Clinical Research, Shanghai General Hospital, Shanghai Jiao Tong University School of Medicine, Shanghai, China; Department of Biochemistry and Molecular Biology, School of Basic Medical Sciences, Capital Medical University, Beijing, China

**Keywords:** prostate cancer, AGGF1, selenium nanomaterials

## Abstract

**Background and Objective:**

Advances in nanomedicine have introduced promising strategies for enhancing drug bioavailability, tumor targeting, and controlled release. This study aimed to investigate the regulatory role of angiogenic factor with G patch and FHA domains 1 (AGGF1) in prostate cancer progression and to evaluate the therapeutic potential of a double-layered silica-shelled selenium nanoparticle (Se@SiO_2_@mSiO_2_) targeting the AGGF1-associated signaling network.

**Methods:**

We performed bioinformatics analyses based on the TCGA-PRAD dataset, including differential expression analysis, WGCNA, and functional enrichment, to identify AGGF1-related pathways. Clinical specimens from 54 prostate cancer patients were collected for validation through Western Blot and immunohistochemistry. We synthesized Se@ SiO_2_@mSiO_2_ *via* a double-layer coating technique and characterized its physicochemical properties by TEM, XRD, XPS, and selenium release assays. To assess the anti-tumor effects of Se@SiO_2_@mSiO_2_, we employed CCK-8, colony formation assays, oxidative stress detection, and established both subcutaneous and orthotopic tumor models in BALB/c nude mice. We further conducted ChIP-qPCR and gene overexpression/silencing experiments to delineate the upstream-downstream relationship between AGGF1 and the Wnt/β-catenin pathway.

**Results:**

AGGF1 was significantly overexpressed in prostate cancer tissues compared with adjacent normal tissues, and high AGGF1 expression was associated with advanced tumor stage, higher Gleason score, and poorer disease-free and overall survival. Se@SiO_2_@mSiO_2_ exhibited a sustained selenium release profile and demonstrated effective intracellular uptake. Treatment with Se@SiO_2_@mSiO_2_ inhibited prostate cancer cell proliferation in a concentration-and time-dependent manner, reduced AGGF1 expression, suppressed oxidative damage, and downregulated β-catenin expression. Overexpression of AGGF1 partially reversed the anti-proliferative effects and Wnt/β-catenin pathway inhibition induced by Se@SiO_2_@mSiO_2_, whereas silencing β-catenin did not significantly alter AGGF1 expression, supporting the upstream regulatory position of AGGF1. *In vivo* experiments confirmed that Se@SiO_2_@mSiO_2_ significantly reduced tumor burden without detectable organ toxicity.

**Conclusion:**

AGGF1 functions as an upstream regulator connecting oxidative stress and Wnt/β-catenin signaling in prostate cancer. Se@SiO_2_@mSiO_2_ exerts anti-tumor effects by downregulating AGGF1 and subsequently suppressing both pathways. These findings position AGGF1 as a pivotal therapeutic target and validate Se@SiO_2_@mSiO_2_ as a candidate nanomedicine for precision treatment of prostate cancer.

## Introduction

As the most common malignant tumor in men worldwide, the disease burden of prostate cancer continues to increase, and epidemiological data show that the incidence in developed countries increases at a rate of 2%–10% every year.^[1,2]^ Despite advances in surgical resection, androgen deprivation therapy (ADT), and chemotherapy, treatment outcomes are often compromised by high recurrence rates, severe adverse events, and the inevitable development of castration resistance.^[3, 4, 5, 6]^ These challenges underscore the urgent need to identify novel molecular targets, particularly those involved in modulating the tumor microenvironment, to facilitate the development of more effective therapeutic strategies beyond conventional modalities.

Among emerging molecular candidates, angiogenic factor with G patch and FHA domains 1 (AGGF1) has garnered attention for its pivotal role in modulating the tumor microenvironment, endothelial cell proliferation, and intracellular signaling cross-talk.^[7,8]^ It is worth noting that studies on colorectal cancer and gastric cancer have confirmed that overexpression of AGGF1 can increase tumor proliferation and decrease patients survival by 2.0–3.3 times,^[9,10]^ but there is still a knowledge gap in its molecular mechanism network in prostate cancer. Given the emerging understanding of AGGF1’s role in modulating key oncogenic pathways, there is a pressing need for targeted therapeutic strategies capable of precisely interfering with this molecular network.

Beyond the identification of novel molecular targets, an equally pressing challenge lies in achieving effective and sustained delivery of therapeutic agents. Advances in nanomedicine have introduced promising strategies for enhancing drug bioavailability, tumor targeting, and controlled release. In the early stage, our team successfully synthesized the first-generation nanomaterial, silica nanoparticle selenium (Se@SiO_2_), and successfully proved that it can inhibit tumor proliferation and calcium oxalate kidney stone formation by regulating the oxidative stress or promote wound repair after transurethral resection of prostate (TURP).^[11, 12, 13]^ However, we found that Se@SiO_2_ still has two major technical barriers in the conversion process: First is the drug sudden release phenomenon, which usually releases active ingredients in a short time; Secondly, Se@SiO_2_ is limited by the single layer silica spherical shell, which makes its drug carrying capacity limited. These two deficiencies make it impossible to effectively play a longterm therapeutic role. Therefore, we further improved the synthesis process on this basis, and synthesized double-layer silica spherical shell nano-selenium (Se@SiO_2_@mSiO_2_), and carried out further research on the related mechanism of its proliferation inhibition on prostate cancer. In this study, we employed integrated bioinformatics analysis and experimental validation to elucidate the AGGF1-associated regulatory mechanisms in prostate cancer and to assess the anti-tumor potential of Se@SiO_2_@mSiO_2_ as a candidate nanomedicine. We found that the treatment with Se@SiO_2_@mSiO_2_ reduced AGGF1 expression, suppressed oxidative damage, and downregulated Wnt/β-catenin activity, ultimately inhibiting tumor growth (Figure 1).

**Figure 1:**
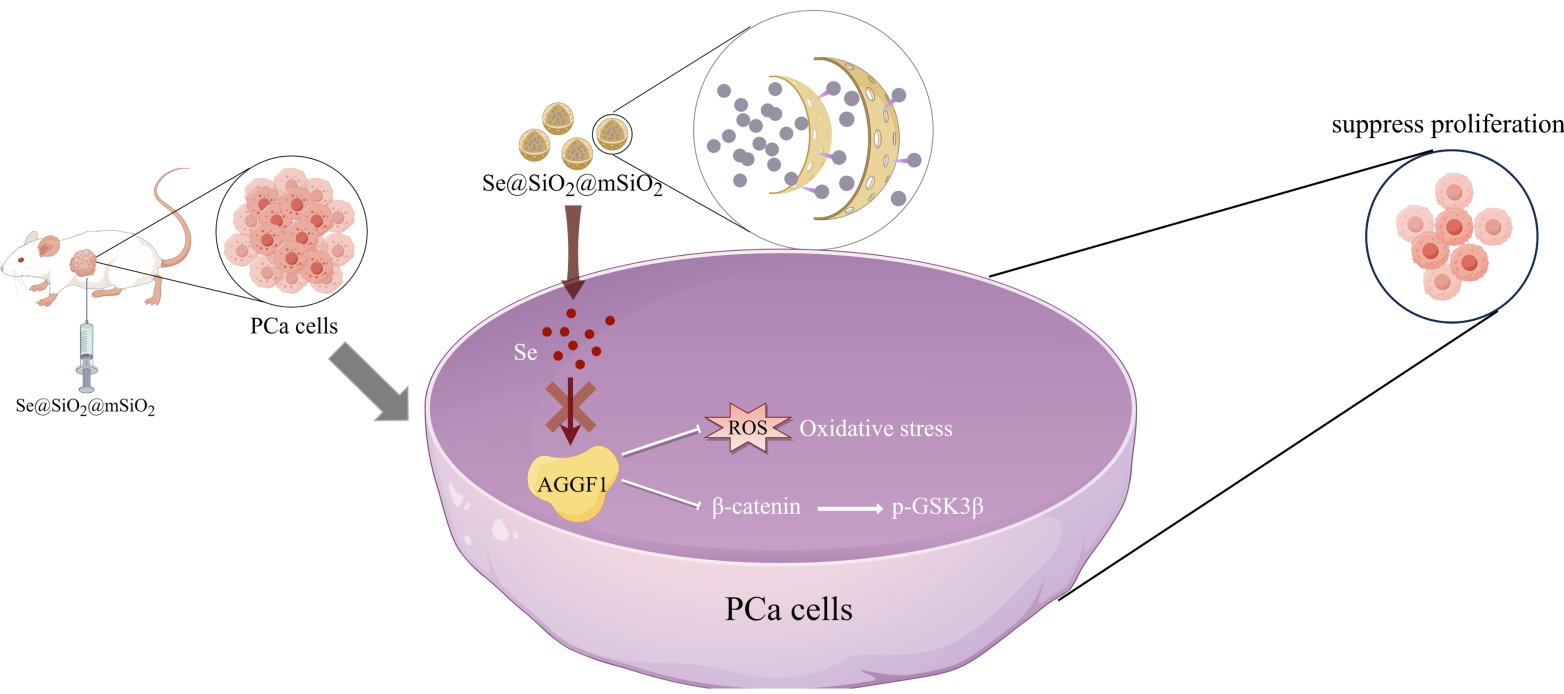
Schematic diagram of the mechanism by which Se@SiO_2_@mSiO_2_ inhibits the proliferation of prostate cancer. Se@SiO_2_@mSiO_2_ releases Se ions layer by layer through different concentration gradients, and further inhibits the Wnt/β-catenin pathway and ROS levels by reducing the expression of AGGF1, thereby suppressing the proliferation of prostate cancer cells. The figure was created with the ''Figdraw'' software.

## Materials and methods

### Synthesis of porous Se@SiO_2_@mSiO_2_ nanospheres

Take 25 mL of deionized water, 15 mL of ethanol, 75 mg of cetyltrimethylammonium bromide and 0.25 mL of ammonia water (28 wt%) and mix them thoroughly. Then add 20 mg of the previously prepared Se@SiO_2_ (thoroughly washed) and stir for 30 min. Drop in 0.12 mL of tetraethyl orthosilicate and stir for 6 h. Centrifuge and wash with water and ethanol. Centrifuge and wash the obtained product with water and ethanol, then place it in 20 mL of water solution (containing 0.2 g of PVP) and stir for 1 h. Then stop stirring, heat to 95°C and maintain for 1 h. Finally, centrifuge and wash with water and ethanol to obtain the Se@SiO_2_@mSiO_2_ nanomaterials. The Se@SiO_2_@mSiO_2_ nanomaterials were characterized by transmission electron microscopy (TEM), X-ray diffractometer (XRD) and X-Ray Photoelectron Spectroscopy (XPS). After that, granular Se@SiO_2_@mSiO_2_ nanoparticles were dissolved in phosphate buffer saline (PBS) solution with pH 7.4 to prepare for the subsequent experiment. Then porous Se@SiO_2_@mSiO_2_-NH_2_ were harvested by centrifugation and washed with ethanol three times. 20 mg porous Se@SiO_2_@mSiO_2_-NH_2_ nanospheres were mixed with 10 mL ethanol, and 1 mg Fluorescein isothiocyanate (FITC) was added under magnetic stirring for 6 h in dark. Fluorescence staining was performed as described previously ^[14]^ to detect the entry of Se@SiO_2_@mSiO_2_-FITC nanospheres into prostate cancer cells. In the case of agitation, the supernatant was centrifugally collected at a preset time, and the Se concentration was determined by Inductively Coupled Plasma Mass Spectrometry (ICP-MS) to obtain the release rate curve of Se.

In terms of the subsequent research grouping, this study did not set free selenium ions as a parallel pharmacological control group. The decision was based on the following scientific fact: in *in vitro* cell experiments, the effective pharmacological concentration of free selenium ions overlaps significantly with the concentration range that causes significant cytotoxicity. Within this range, any observed inhibition of cell proliferation is mainly attributed to non-selective oxidative stress-induced cell death, rather than specific regulation of a certain pathway. Therefore, it is impossible to determine a concentration that ensures high cell survival rate while also demonstrating specific pharmacological activity, thus making direct biological efficacy comparisons unjustified. This study aims to evaluate the specific biological effects of S Se@SiO_2_@mSiO_2_ as a novel nanodelivery system, whose mechanism of action has been confirmed to be related to the regulation of specific signaling pathways. The dose used in the experiments for Se@SiO_2_@mSiO_2_ is far lower than the dose range typically associated with significant toxicity when free selenium compounds cause equivalent antitumor effects in the literature. The unique advantages of Se@SiO_2_@mSiO_2_ are demonstrated through its core-shell structure’s sustained-release property, enhanced intracellular accumulation, and its association with the specific molecular mechanism in this study.

### Bioinformatics analysis

The TCGA-PRAD dataset (GDC Data Release 38.0, including 499 primary prostate adenocarcinoma samples and 52 paired normal tissues) was analyzed using R v4.3.1 under the following workflow.

#### Data preprocessing

Raw RNA-seq counts were normalized using the DESeq2 median-of-ratios method, with batch correction performed *via* the ComBat algorithm in the sva package to address technical variability. Genes with low expression (FPKM < 1 in > 90% of samples) were excluded prior to downstream analysis.

#### Differential expression analysis

Paired differential analysis (*n* = 52 tumor-normal pairs) was implemented using the limma package (v3.58.1). A linear model with empirical Bayes moderation was applied, with significance defined as Benjamini-Hochberg-adjusted *P* < 0.05 and |log2 (fold change)| > 1. Volcano plots were generated using ggpubr (v0.6.0).

#### WGCNA network construction

Co-expression networks were built using WGCNA (v1.72-5).

Soft threshold power selection: β = 11 achieved scale-free topology (scale-free fit index R^2^ = 0.90, slope = -2.3).

Module detection: Dynamic hybrid tree cutting (deep Split= 2, min Cluster Size = 50) identified 18 co-expression modules.

Module preservation: > 85% of modules showed significant preservation (*Z* summary > 10) in 1000 bootstrap iterations.

Trait association: Module eigengene-trait correlations were tested using two-sided Pearson’s test with Bonferroni correction.

#### Functional enrichment

GO/KEGG analyses used cluster Profiler (v4.10.0).

Background gene set: 15,342 protein-coding genes from GENCODE v44.

Enrichment thresholds: Adjusted *P* < 0.05 (Benjamini-Hochberg method has been used to control the false discovery rate).

Semantic simplification: Redundant terms were removed using simplify Enrichment (v1.10.0) with Jaccard similarity < 0.5.

#### Statistical reporting

All p-values underwent multiple testing correction using the Benjamini-Hochberg procedure unless otherwise specified. Visualization color schemes followed Nature Portfolio guidelines using ggsci (v3.0.0) palettes.

### Patients and tissue specimens

This study included tissue samples from 54 patients with pathologically diagnosed prostate cancer who were admitted to the Department of Urology, Beijing Friendship Hospital Affiliated to Capital Medical University. All individuals involved in the study signed informed consent and the study was conducted in accordance with the Declaration of Helsinki of the World Medical Association. All patient-derived specimens were collected and archived in accordance with the protocols approved by the BioEthics Committee of Beijing Friendship Hospital Affiliated to Capital Medical University, and the relevant procedures were carried out in accordance with the approved guidelines. Based on the existing resources of the prostate cancer biobank at Beijing Friendship Hospital, the 54 cases are complete cases that met the inclusion criteria during the same period. The TNM stage, Gleason score, Disease-Free Survival (DFS) and Overall Survival (OS) of all patients were collected for follow-up analysis. In all patients, tumor tissues and the paired adjacent normal tissues were collected after radical prostatectomy, and then frozen in liquid nitrogen, or immobilized in formalin and embedded in paraffin for subsequent assays.

### Western blot analysis

After harvesting proteins, proteins with different molecular weights were separated and trans-transferred to PVDF membrane by electrophoresis and electrotransfer. After blocking with NcmBlot blocking buffer (P30500, NCM), membrane and anti-AGGF1 (11889-1-AP, Proteintech), β-catenin (51067-2-AP, Proteintech), p-GSK3β (14850-1-AP, Proteintech), GSK3β (67329–1-Ig, Proteintech), β-actin (66009-1-Ig, Proteintech) and GAPDH (60004-1-Ig, Proteintech) were incubated overnight at 4 °C. Next, the membrane was incubated with secondary antibodies and visualized with enhanced chemiluminescence (ECL). β-actin or GADPH were used as loading controls.

### Immunohistochemical staining

The expression of AGGF1 in tissues and cell lines was detected by immunohistochemical staining described in previous studies.^[11]^ The staining degree was scored according to the percentage of positive staining cells: low expression (≤50%), high expression ( > 50%). The corresponding primary antibody was Ki67 (27309-1-AP, Proteintech; 28074–1-AP, Proteintech) and AGGF1 (11889-1-AP, Proteintech).

### Animal experiments

In this study, healthy male BALB/c nude mice (8-week-old) were selected, which were housed in the animal experiment center of Capital Medical University Beijing Friendship Hospital under pathogen-free conditions. An animal model of prostate cancer was constructed by subcutaneous injection of 1 × 10^7^ prostate cancer cells (22RV1) once after androgen castration surgery.

As for the *in situ* tumor formation model of prostate cancer, we employed the *in situ* injection surgery method for modeling. The mice were induced and maintained under anesthesia by inhaling isoflurane (2%). After confirming the depth of anesthesia, they were placed in a prone position on a sterile operating table. The lower back and the area around the ribs were shaved using a depilator, and the surgical area was disinfected three times alternately with iodophor and 75% ethanol. Make a longitudinal skin incision about 1 cm long along the midline. Delicate dissection of the subcutaneous tissue and muscle layer is performed to expose the peritoneum. Using sterile cotton swabs, gently push the intestinal tube to one side. Below the bladder and seminal vesicle gland, a pale yellow, lobulated prostate dorsal lobe can be seen. Draw 50 μL of the cell suspension. At the middle part of the dorsal lobe of the prostate, insert the needle at an angle of approximately 30 degrees diagonally into the prostate tissue, with a depth of about 2–3 mm. Slowly inject the cell suspension. After the injection is completed, keep the needle in the original position for 1 min, then slowly withdraw it to prevent the backflow of the cell suspension. Discontinuously suture the muscles and the wound, and disinfect the wound again with iodophor. Place the mice on a 37°C warming pad until they fully regain consciousness, then return them to the cage for observation. For three consecutive days after the surgery, the activity, diet, weight and wound healing of the mice were observed daily. Subsequently, the mice were weighed twice a week and the presence of neurological compression symptoms such as urinary difficulty and hind limb paralysis was closely monitored. At the end of the experiment, the mice were sacrificed. The tumors were completely removed, weighed, fixed with 4% paraformaldehyde, and embedded in paraffin for H & E staining and histopathological analysis to confirm the formation of prostatic *in situ* tumors.

The two model mice were equally divided into the control group and Se@SiO_2_@mSiO_2_ group. Meanwhile, Se@SiO_2_@mSiO_2_ group were injected with Se@SiO_2_@mSiO_2_ solution intraperitoneally on 1^st^ day (1 mg/ kg) only. The dose of the Se@SiO_2_@mSiO_2_ was determined based on the safety and efficacy results of our team’s previous research on this series of nanomaterials to ensure the goodness *in vivo* biocompatibility. Control group were injected with the same amount of normal saline. On the 14^th^ day, the mice were sacrificed, and serum, tumor tissue, heart, spleen, lung, liver and kidney tissue were collected for follow-up experiments. All experiments above were approved by the Animal Care and Use Committee of Capital Medical University (No. 2024-P2-438-01).

### Cell culture and transfection

Prostate cancer cells (22RV1 and C4–2B) were purchased from Pricella (CL-0004) and Mlbio (ml096023), respectively. All cells were cultured in a humidified atmosphere containing 5%CO_2_ at 37 °C. Both cells were cultured in Roswell Park Memorial Institute 1640 (RPMI-1640 with HEPES and PhenolRed) medium containing 10% fetal bovine serum (non-Activated Charcoal Absorbed FBS) and 1% penicillin/streptomycin.

To construct overexpression of AGGF1 in prostate cancer cells, the PCR product (GenBank Accession No. BC002828) covering the open reading frame of AGGF1 was cloned into the pcDNA3.1 vector. The specific primers used are as follows (AGGF1-F: 5′-GGA CTA GTT TGC GAA AAC ATG GGT AGT G-3′; AGGF1-R: 5′-CCC AAG CTT TGC CTG CAA TGG TCT TTA TC-3′). The final recombinant plasmid was named pcDNA3.1-AGGF1. This study was based on the previously published effective sequence design for β-catenin siRNA. The siRNA sequence used as follows: β-catenin-F: 5′-ACGGAGGAAGGTCTGAGGAG-3′; β-catenin-R: 5′- AGCCGCTTTTCTGTCTGGTT-3′. A non-targeted sequence was used as the normal control (NC). By culturing in a final concentration of 2 μg/mL puromycin, PCa cells with reduced endogenous AGGF1 expression were screened out. Lipofectamine 3000 was used to transfect PCa cells following the manufacturer’s instructions.

### Cell proliferation inhibition

22RV1/C4-2B cells were placed into 6-well cultural plates, and incubated with Se@SiO_2_@mSiO_2_ in different concentration or duration. After processing, the cells were digested with trypsin, centrifuged for collection, and then resuspended in binding buffer for subsequent experiments. The inhibitory effect of Se@SiO_2_@mSiO_2_ on prostate cancer cells under different treatment conditions was assessed by Cell Counting Kit-8 (ab228554, abcam) and colony formation assay.

### Detection of oxidative stress

Oxidative stress level of prostate cancer cells after Se@SiO_2_@mSiO_2_ treatment was evaluated. Oxidative factors included catalase (CAT, S0051, Beyotime), malondialdehyde (MDA, K2167, APExBIO), reduced glutathione (GSH, S0052, Beyotime) and oxidized glutathione disulfide (GSSG, S0053, Beyotime) were all detected by available assay kits. The experimental procedures strictly follow the descriptions in the instruction manual. The intensity of the ultraviolet absorption signal at the corresponding wavelength was detected by ultraviolet microplate reader, and then the contents of different indicators were calculated.

### Chromatin immunoprecipitation quantitative real-Time PCR (chip-qpcr) assays

ChIP Assay Kit (Beyotime, Shanghai, China) has been used according to the manufacturer’s protocol. DNA was immunoprecipitated after the sonicated celllysates using anti-AGGF1 antibodies (11889-1-AP, Proteintech) diluted at a ratio of 1: 2000 at 4°C and treated for 12 h; IgG (30000-0-AP, Proteintech, 1: 2000) served as the negative control. The AGGF1 combined DNA was subjected to following quantitative Real-Time PCR assay. The primer sequences were as follows: β-catenin-F: 5′-ACGGAGGAAGGTCTGAGGAG-3′; β-catenin-R: 5′-AGCCGCTTTTCTGTCTGGTT-3′; GAPDH-F: 5′-GGG AAG GTG AAG GTC GGA GT-3′; GAPDH-R: 5′-GGG GTC ATT GAT GCRC AAC A-3′. The specificity of all primers was verified by standard PCR, and the results confirmed the presence of a single amplified product of the expected size.

### Statistical analysis

The statistical analysis software used was R software (version 4.3.0) and GraphPad Prism 9. All data are presented in the form of mean ± standard deviation. The Cox proportional hazards model was used to evaluate the OS and DFS of the patients in the two groups. Student’s t-test was used for the comparison of data between two groups. When analyzing whether there are overall differences in the means of quantitative data among multiple groups, Analysis of Variance (ANOVA) is first employed. If ANOVA is significant (*P* < 0.05), further analysis of specific pairwise differences between groups is conducted through post-hoc tests. All results are considered statistically significant if *P* < 0.05.

## Result

### AGGF1 is closely related to the malignancy degree and prognosis of prostate cancer

In this study, through the analysis of TCGA and GTEx databases, it was found that the expression level of AGGF1 in prostate cancer tissues was significantly higher than that in normal tissues (Figure 2A). Meanwhile, among patients with pathological Gleason score ≥8, AGGF1 levels were also higher (Figure 2B). We also further collected clinicopathologic specimens of prostate cancer patients, and divided them into high AGGF1 group and low AGGF1 group according to the difference of its expression level. Through clinical data analysis, it was found that, The tumor risk grade, T stage and Gleason score in the high AGGF1 expression group were significantly higher than those in the low AGGF1 expression group (Figure 2C–D), and the differences were statistically significant (*P* < 0.05). Further Western Blot and immunohistochemical results showed that the content of AGGF1 in the patient’s tumor tissues was significantly higher than that in the adjacent tissues (Figure 2E–F), which further confirmed the relationship between AGGF1 and the malignancy of prostate cancer. Meanwhile, in the data analysis of clinical patients, we also found that the DFS (Figure 2G) and OS (Figure 2H) of patients in the group with high AGGF1 expression were both shorter than those in the group with low AGGF1 expression, and this result was also supported by the analysis of the contents of the TCGA database (Figure 2I). These results indicate that AGGF1 may play a crucial role in the occurrence and development of prostate cancer, and high expression of AGGF1 may predict a higher degree of malignancy and a worse prognosis of patients.

**Figure 2:**
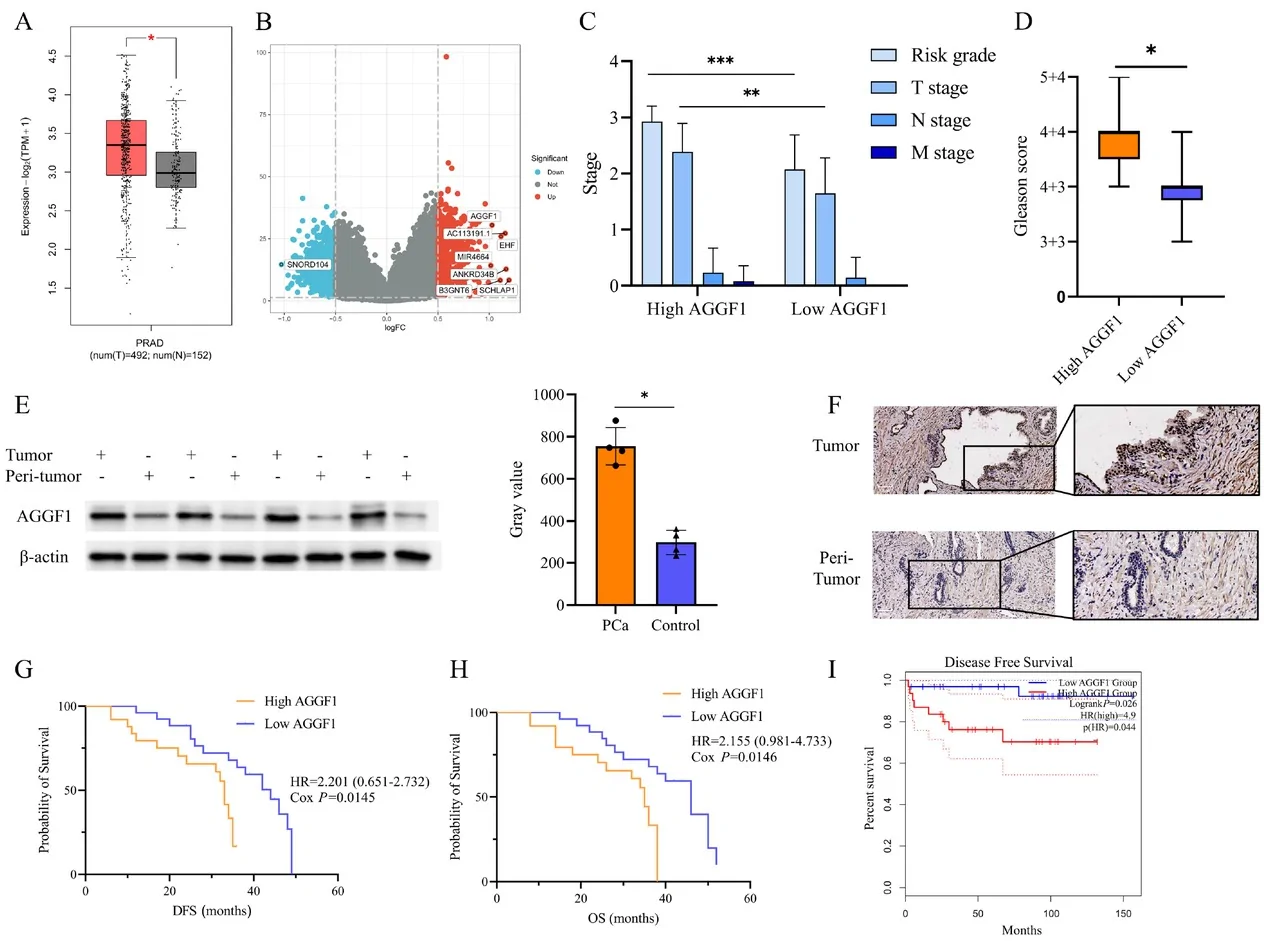
Correlation of AGGF1 with prostate cancer malignancy and prognosis. (A) The difference of AGGF1 content between tumor tissue and normal tissue was analyzed by TCGA and GTEx databases; (B) The expression difference of AGGF1 among patients with different Gleason scores was analyzed by TCGA database; (C) The difference of tumor risk grade and TNM stage among PCa patients with different AGGF1 expression levels; (D) The differences of Gleason scores in PCa patients with different AGGF1 expression levels; (E) Western Blot analysis of AGGF1 protein expression between prostate cancer tissues and adjacent tissues; (F) Immunohistochemical detection of AGGF1 levels in prostate cancer and para-cancerous tissues; (G) DFS differences among PCa patients with different AGGF1 expression levels; (H) Differences in OS among PCa patients with different AGGF1 expression levels; (I) The differences in DFS among PCa patients with different AGGF1 expression levels were analyzed using the TCGA database. ^*^P < 0.05, ^**^P < 0.01, ^***^P < 0.001.

### Correlation between the Wnt/β-catenin pathway and AGGF1 and oxidative stress in prostate cancer

In terms of bioinformatics analysis, this study further explored the interaction between AGGF1 and other pathways in prostate cancer by constructing and selecting the appropriate WCGNA module (Figure 3A–B). GO and KEGG enrichment analysis showed that the Wnt/β-catenin pathway was highly enriched in patients with high AGGF1 expression (Figure 3C-D). Meanwhile, in patients with high AGGF1 expression, CTNNB1 (β-catenin) and GSK3B, key proteins in the Wnt/β-catenin pathway, also showed high expression, and there was a significant linear correlation (Figure 3E–H). We further analyzed the correlation between the Wnt/β-catenin pathway and other pathways in prostate cancer. GSEA (Figure 4A), GO (Figure 4B-C) and KEGG (Figure 4D) analysis showed that the oxidative stress pathway was enriched in the group with high expression of GSK3B and CTNNB1.

**Figure 3:**
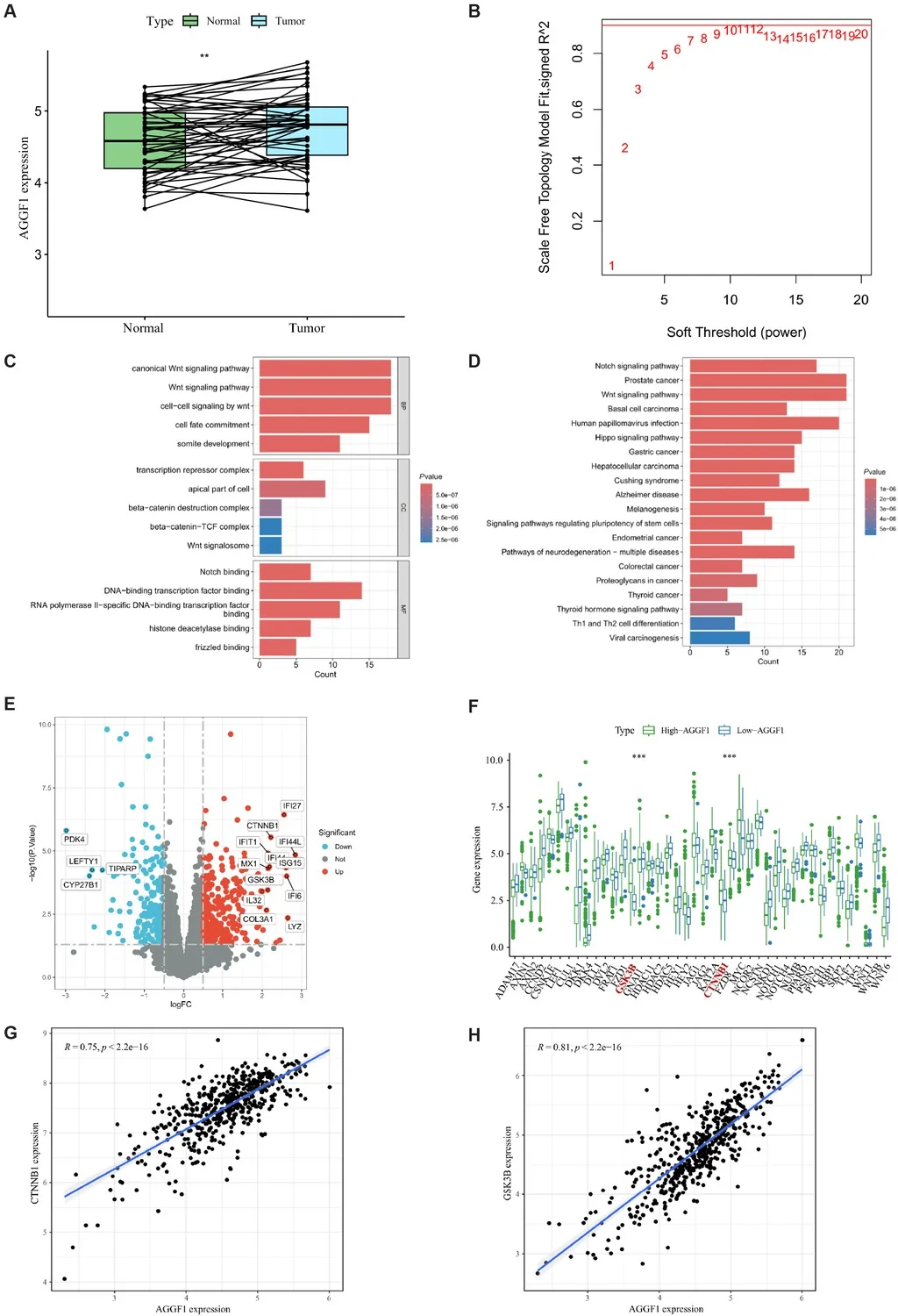
Bioinformatics analysis of the AGGF1-Wnt/β-catenin regulatory network in prostate cancer. (A) The expression difference of AGGF1 between prostate cancer tissues and adjacent tissues in TCGA database; (B) Determination of soft threshold in WGCNA network analysis; (C) Functional Enrichment Analysis of 228 Overlapping Genes: KEGG Pathway and WNT Signaling - Related Gene Ontology (GO) Terms; (D) Functional Enrichment Analysis of 228 Overlapping Genes: KEGG Pathway and Disease - Associated Terms; (E) Volcano plot showing differentially expressed genes between different AGGF1 expression group; (F) Box plot of gene expression differences in different AGGF1 expression groups; (G) The relationship between the expression level of the CTNNB1 and AGGF1; (H) The relationship between the expression level of the GSK3B and AGGF1.

**Figure 4:**
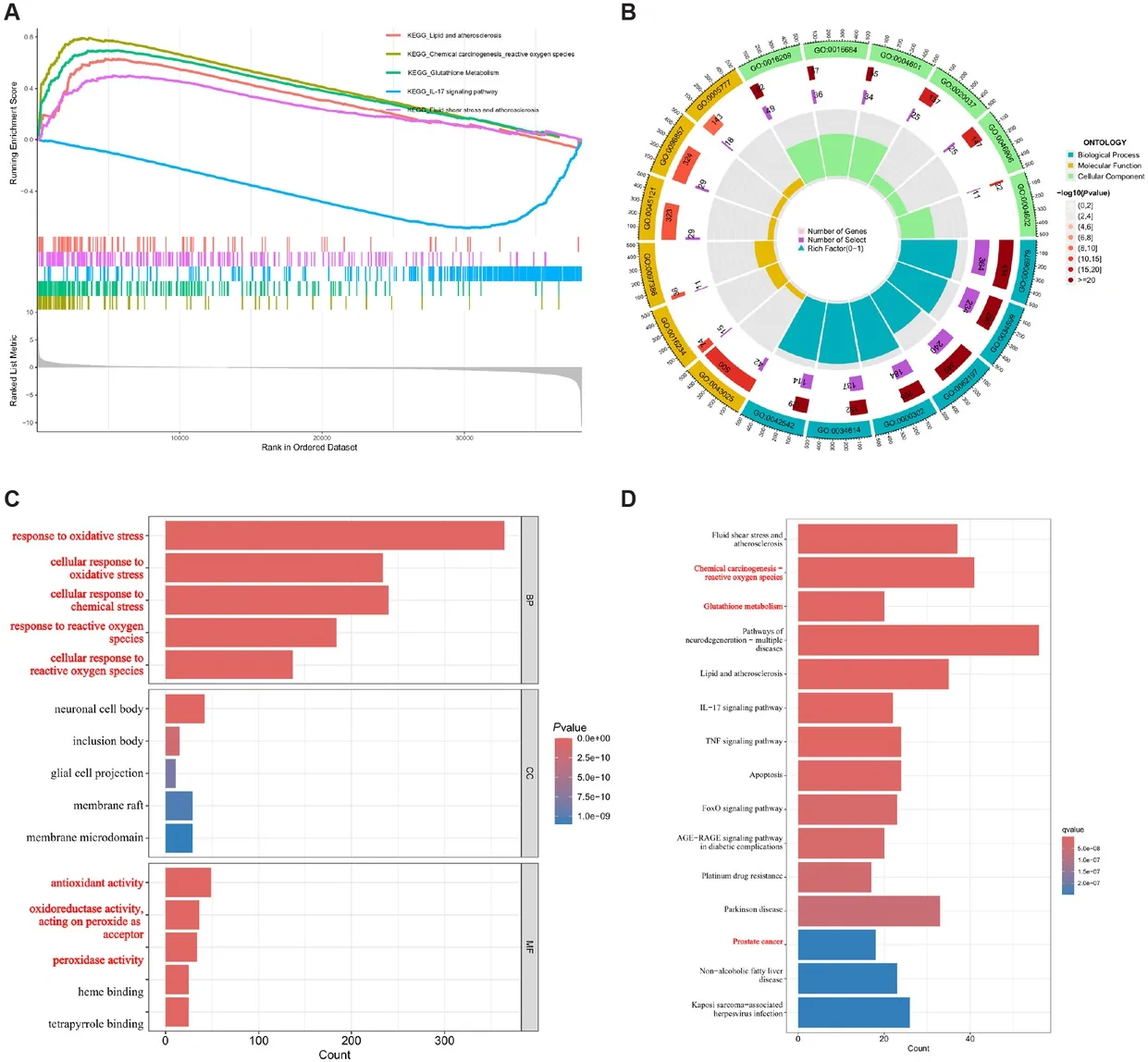
Bioinformatics analysis of the correlation between Wnt/β-catenin pathway and ROS. GSEA (A), GO (B–C) and KEGG (D) enrichment analysis in groups with different GSK3B and CTNNB1 expression levels.

### Synthesis and identification of double-layer silica nano-selenium materials (Se@SiO_2_@mSiO_2_)

Previously, our team has successfully synthesized a single layer of silica nano-selenium material (Se@SiO_2_), and successfully proved that it can inhibit the formation of calcium oxalate crystals by regulating the oxidative stress level of cells ^[12]^ or promote wound repair after prostate surgery.^[13]^ Unfortunately, we found that Se@SiO_2_ had limited drug loading and could not reposefully release selenium ions in a long time. Therefore, we further adjusted the production technology and process of nanomaterials on the basis of Se@SiO_2_ and successfully prepared double-layer silica nano-selenium material (Se@SiO_2_@mSiO_2_) (Figure 5). The phase structure of porous Se@SiO_2_@mSiO_2_ nanospheres was inspected by TEM (Figure 6A–B) and XRD(Figure 6C) pattern, and several well-defined characteristic peaks demonstrate standard hexagonal phase selenium. Further detection results of selenium ion release rate indicate that, compared with Se@SiO_2_, Se@SiO_2_@mSiO_2_ has a slower and more stable selenium release rate (Figure 6D), which also enables it to play a longer therapeutic role. As shown in XPS spectra (Figure E), in the Se 3 d spectrum, the peak at 54.62 eV corresponds to the 3 d5/2 orbital of selenium in the 0-valence state, while the peak at 60.03 eV corresponds to the 3 d3/2 orbital of selenium in the 0-valence state. In the Si 2p spectrum, the peaks at 102.92 eV and 103.01 eV both correspond to silicon in the +4-valence state. In the O 1 s spectrum, the peaks at 532.37 eV and 531.13 eV both correspond to oxygen in the-2-valence state. FITC was also applied to Se@SiO_2_@mSiO_2_ to observe their distribution in cells. We had successfully observed under confocal microscopy that Se@SiO_2_@mSiO_2_ could effectively enter prostate cancer cells and distribute relatively evenly in the cytoplasm (Figure 6F). To comprehensively assess the biocompatibility profile, we conducted systematic histopathological evaluation of vital organ systems (*via* H & E staining) coupled with serological profiling of critical biomarkers. The results indicated that the experimental dose of Se@SiO_2_@mSiO_2_ used in this study maintained structural integrity in tissues of nude mice (Figure 6G). Furthermore, pivotal organ functions remained unaffected (Table 1).

**Figure 5:**
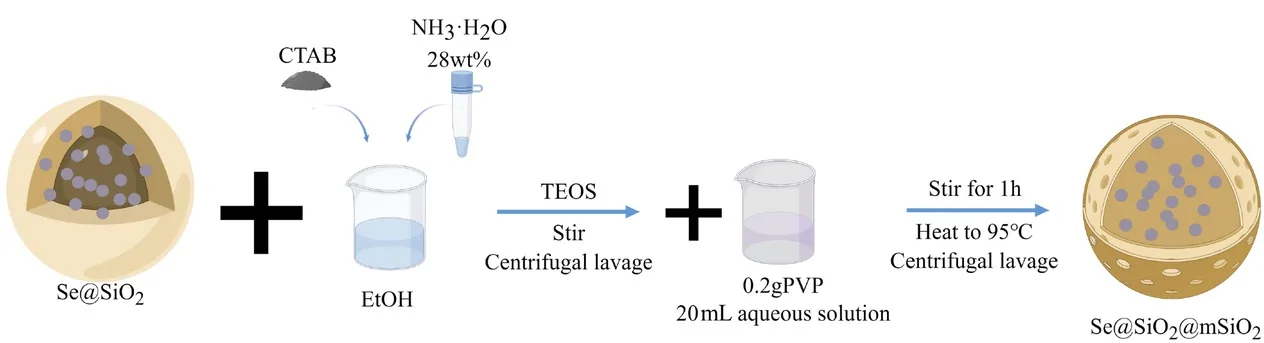
Se@SiO_2_@mSiO_2_ preparation process.

**Figure 6:**
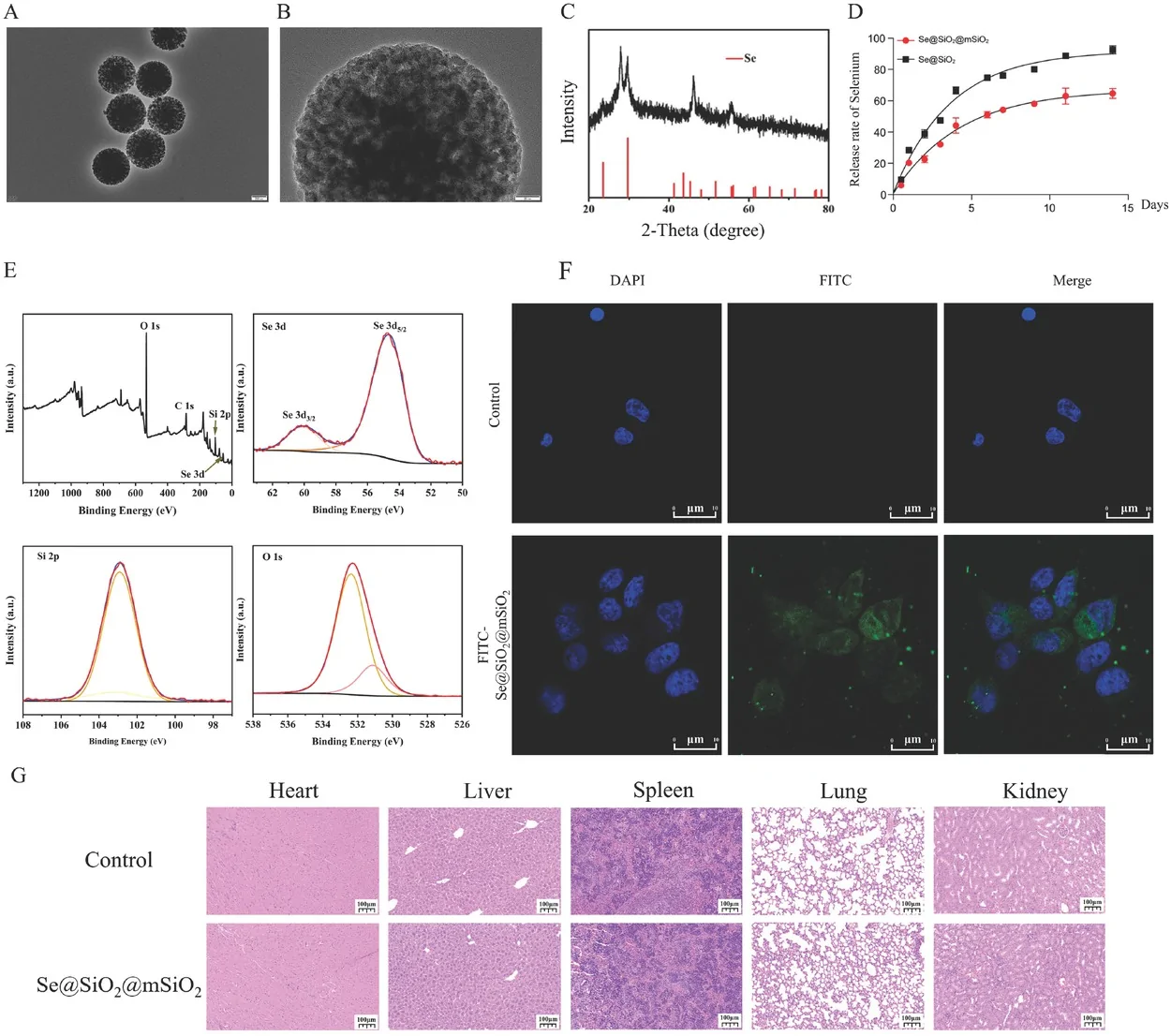
Material science identification of Se@SiO_2_@mSiO_2_. (A) TEM characterization of the sphere-shell structure Se@SiO_2_@mSiO_2_; (B) TEM image of the structure of Se@SiO_2_@mSiO_2_ nanospheres; (C) XRD patterns of Se@SiO_2_@mSiO_2_ and standard Se hexagonal phase (JCPDS card number: 65-1876); (D) Comparison of Se release rate between Se@SiO_2_@mSiO_2_ and Se@SiO_2_; (E) XPS analysis of Se@SiO_2_@mSiO_2_ (Full spectrum, Se 3 d, Si 2p, O 1 s.); (F) The distribution of FITC-Se@SiO_2_@mSiO_2_ in cells was observed under confocal microscope; (G) Biosafety test for Se@SiO_2_@mSiO_2_.

**Table 1:** Effects of Se@SiO_2_@mSiO_2_ on pivotal organ functions in subcutaneous tumor model nude mice.

Parameters	Control (*n* = 6)	Se@SiO_2_@mSiO_2_ (*n* = 6)	*P* value
ALT (μg/L)	12.33 ± 0.50	11.72 ± 1.43	0.522
AST (μg/L)	40.18 ± 1.26	40.12 ± 3.20	0.978
LDH (μg/L)	175.42 ± 32.39	181.13 ± 22.34	0.814
CKMB (μg/L)	4.35 ± 0.17	4.41 ± 0.50	0.863
TB (μg/mL)	1.59 ± 0.08	1.59 ± 0.30	0.997
Cr (μg/mL)	2.74 ± 0.22	2.42 ± 0.28	0.197
ALB (Log_10_, μg/mL)	4.58 ± 0.03	4.59 ± 0.02	0.648

ALT: alanine aminotransferase; AST: aspartate aminotransferase; LDH: lactate dehydrogenase; CKMB: creatine kinase-MB; TB: total bilirubin; Cr: creatinine; ALB: albumin.

### Se@SiO_2_@mSiO_2_ can effectively inhibit the proliferation of prostate cancer

We further verified the inhibitory effect of Se@SiO_2_@ mSiO_2_ on the proliferation of prostate cancer through *in vivo* and *in vitro* experiments. First, we detected the inhibitory effect of Se@SiO_2_@mSiO_2_ on prostate cancer cell line (22RV1/C4-2B) and epithelial cell lines of benign prostatic hyperplasia tissue (BPH-1) at different concentrations through CCK8 *in vitro*. The results suggest that with the increase of nanomaterial action concentration, its inhibitory effect on the proliferation of prostate cancer cells was significantly higher than that of hyperplasia prostate epithelial cells (Figure 7A), and with prolonged action time, Se@SiO_2_@mSiO_2_ also had a more obvious inhibitory effect on prostate cancer cells (Figure 7B). Meanwhile, the results of plate cloning formation experiment also confirmed that Se@SiO_2_@mSiO_2_ could effectively inhibit proliferation of prostate cancer cells (Figure 7C). *In vivo* animal experiments, we found that the tumor weight of nude mice treated with Se@SiO_2_@mSiO_2_ was significantly lower than that of the control group (Figure 7D), which also confirmed the effectiveness of Se@SiO_2_@mSiO_2_ in treatment of prostate cancer.

**Figure 7:**
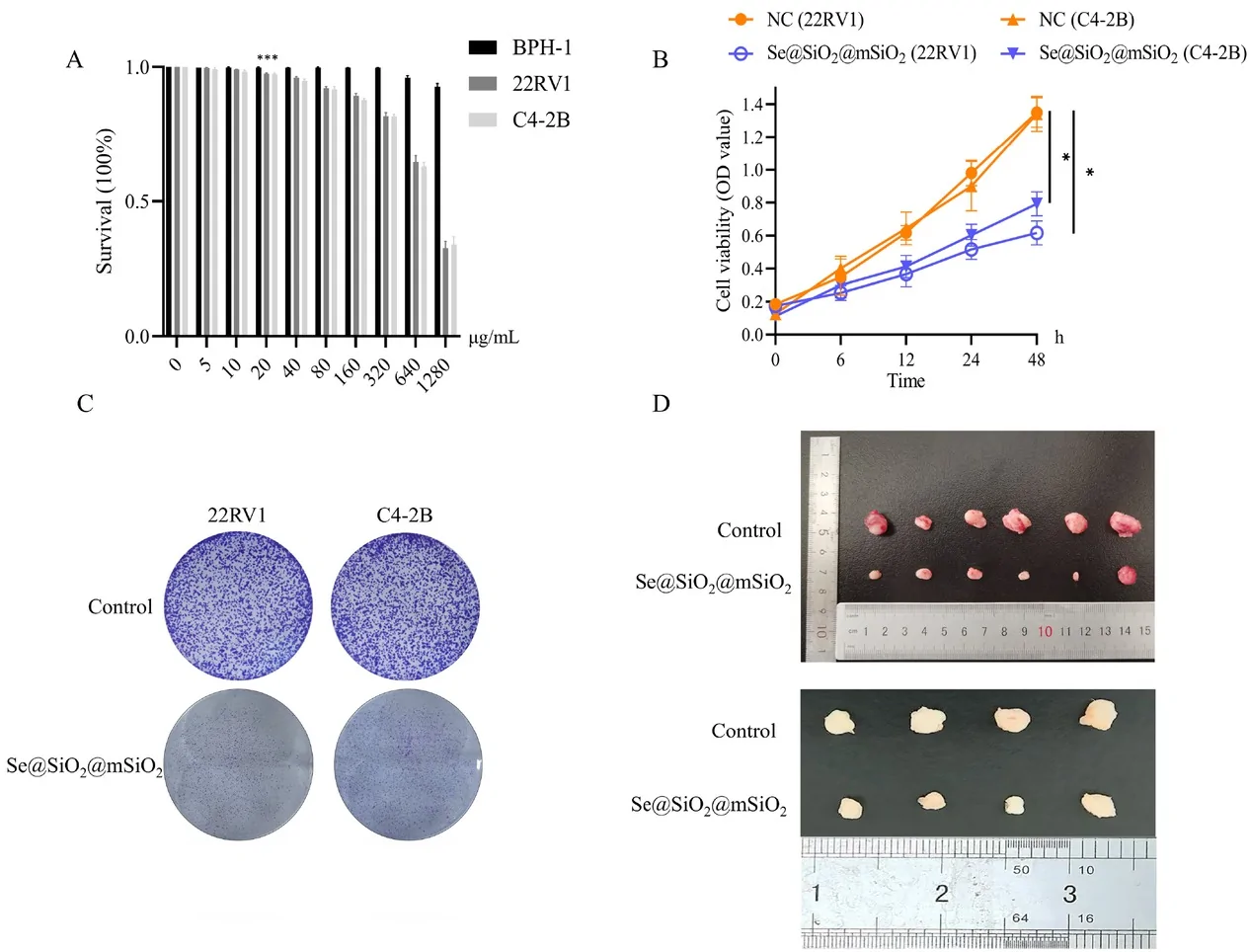
Epigenetic identification of Se@SiO_2_@mSiO_2_ inhibiting the proliferation of prostate cancer cells. (A) Toxicity of Se@SiO_2_@mSiO_2_ at different concentrations to prostate cancer cells (22RV1, C4-2B) and epithelial cell lines of benign prostatic hyperplasia tissue (BPH-1); (B) The effects of different Se@ SiO_2_@mSiO_2_ treatment duration on the proliferation of prostate cancer cells (22RV1, C4-2B) were detected by CCK8; (C) The effects of Se@SiO_2_@mSiO_2_ on prostate cancer cells (22RV1, C4-2B) were detected by colony formation assay;(D) Verification of the inhibitory effect of Se@SiO@mSiOon prostate cancer _22_ in BALB/c models (Above: subcutaneous transplantation tumor model; below: *in situ* tumor formation model). ^*^P < 0.05, ^***^P < 0.001.

### The mechanism of Se@SiO_2_@mSiO_2_ inhibits prostate cancer through AGGF1

After further testing the oxidative stress levels of prostate cancer cells in different treatment groups, we were found that Se@SiO_2_@mSiO_2_ could effectively increase the content of catalase in prostate cancer cells and further inhibit oxidative stress (Figure 8A–E). The results of plate cloning experiment showed that the proliferation of prostate cancer cells was more obvious after overexpression of AGGF1 (Figure 9A). Immunohistochemical results of *in vivo* experiments further confirmed that Se@SiO_2_@mSiO_2_ could effectively inhibit the expression of AGGF1 and Ki-67 in tumor tissues (Figure 9B). In addition, Western Blot results showed that Se@SiO_2_@mSiO_2_ could inhibit the expression of β-catenin, a key protein in the AGGF1 and Wnt/β-catenin pathways, and the phosphorylation of GSK3B. Moreover, the expression of β-catenin and phosphorylated GSK3B protein was correspondingly increased after overexpression of AGGF1 (Figure 9C). Furthermore, the ChIP-qPCR results revealed that the ability of AGGF1 protein to bind to β-catenin was reduced when Se@SiO_2_@mSiO_2_ was applied alone, while this phenomenon could be reversed by overexpressing AGGF1 (Figure 9D). In order to further explore the upstream-downstream relationship between AGGF1 and Wnt/β-catenin signal, we also conducted Western Blot assays to detect the expression of AGGF1 after silencing the β-catenin gene. The results showed that the expression of AGGF1 was not significantly affected after inhibition of β-catenin (Figure 9E). Mechanistically, these findings provide biochemical evidence suggesting that AGGF1 potentially functions as an upstream regulatory component of the Wnt/β-catenin signaling cascade.

**Figure 8:**
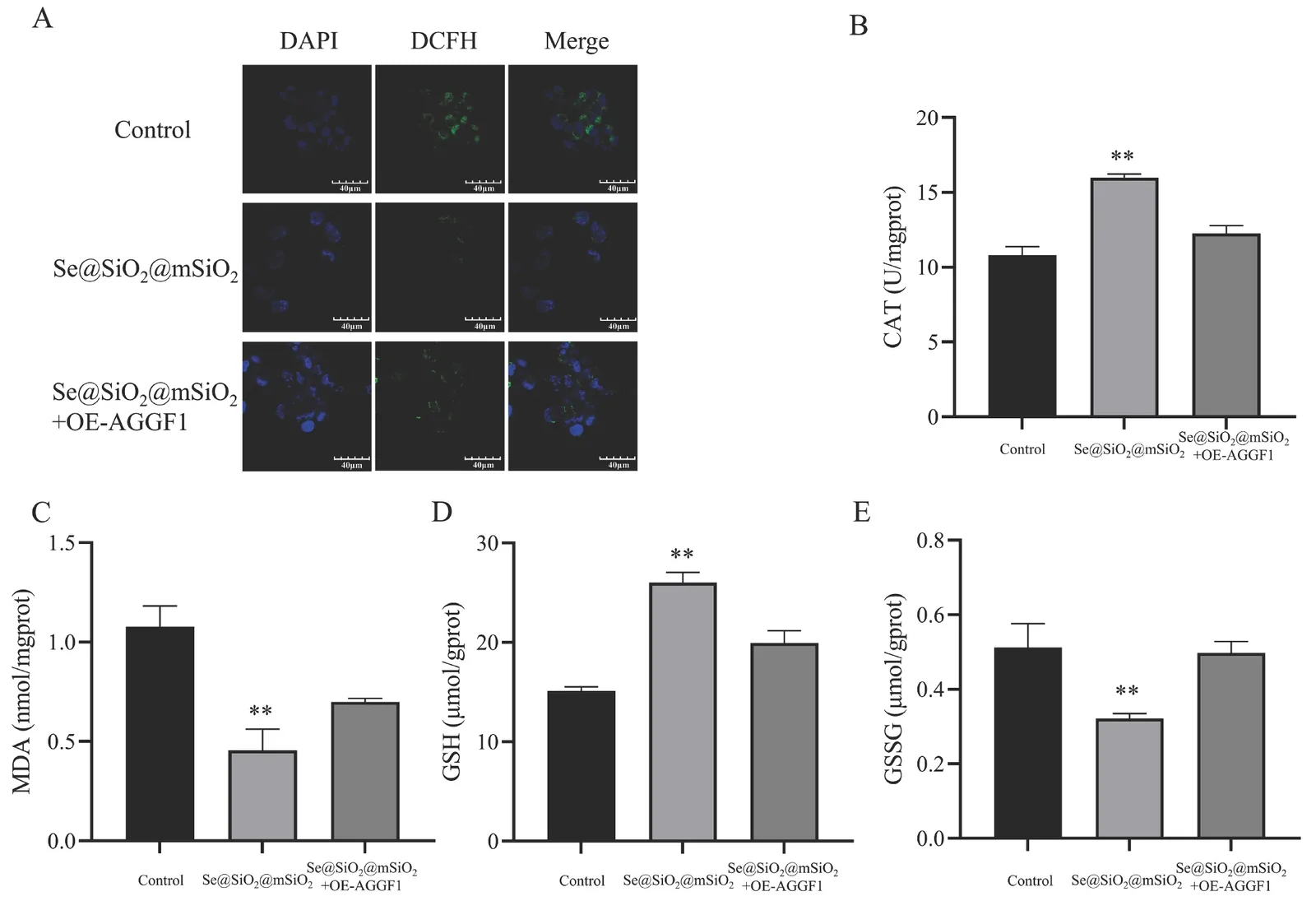
Effects of Se@SiO_2_@mSiO_2_ and AGGF1 on oxidative stress levels in prostate cancer cells. (A) The changes of cellular oxidative stress were observed under confocal microscopy; (B–E) The oxidation status of prostate cancer cells was detected by CAT (B), MDA (C), GSH (D) and GSSG (E) respectively. ^**^P < 0.01.

**Figure 9:**
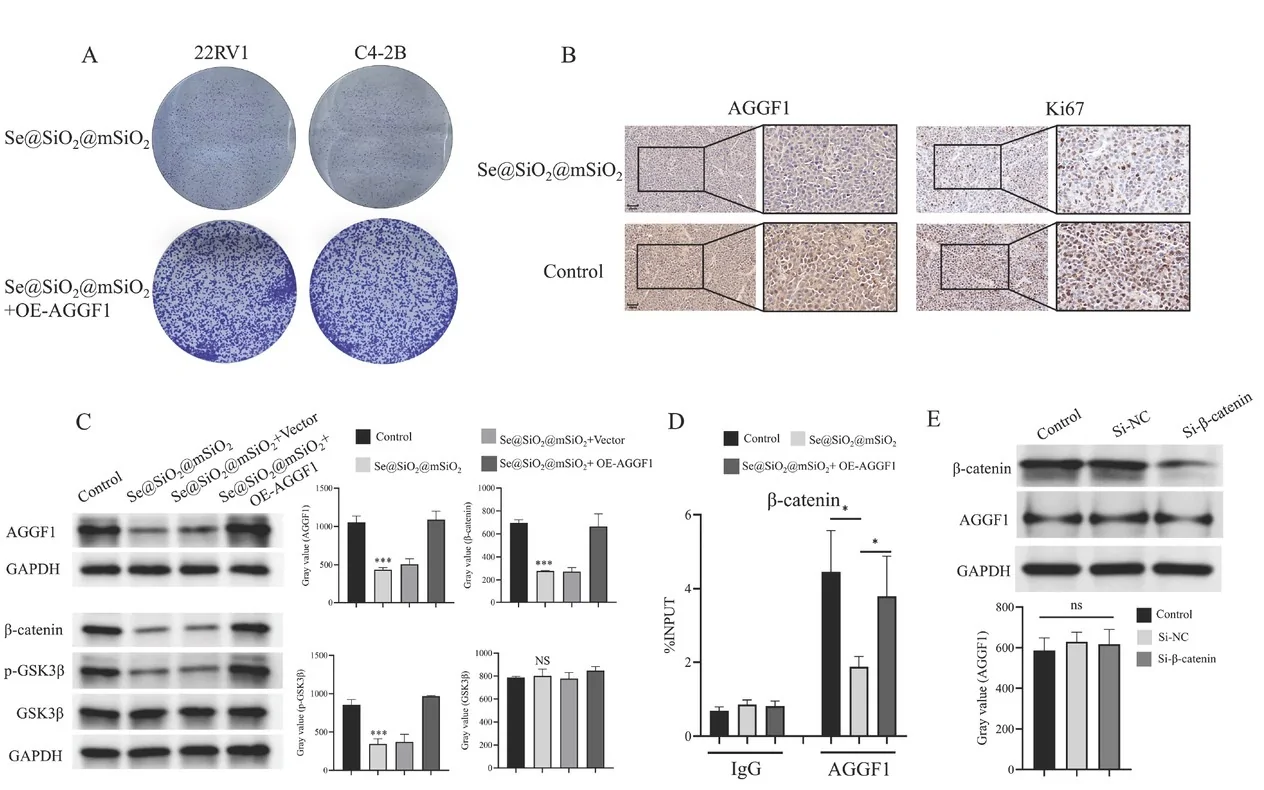
Relationship between AGGF1 and Wnt/β-catenin pathway. (A) Effect of overexpression of AGGF1 on colony formation of prostate cancer cells; (B) The expression of AGGF1 and Ki-67 in BALB/c model was detected by immunohistochemistry; (C) The expression of AGGF1 and key proteins of Wnt/β-catenin pathway in different treatment groups was detected by Western Blot; (D) The binding situation of AGGF1 and β-catenin RNA in different treatment groups was detected by ChIP-qPCR; (E) Western Blot was used to detect the expression levels of AGGF1 and β-catenin proteins after silencing β-catenin. ^*^P < 0.05, ^***^P < 0.001.

## Discussion

In this study, we engineered a dual-layer silica nanoselenium system (Se@SiO_2_@mSiO_2_) to overcome burst release and low drug loading in conventional single-layer nanocarriers. Further avoids the limitations of the existing clinical treatment methods for prostate cancer, which have many side effects and prone to drug resistance.^[15, 16, 17]^ Building on our previous work, this hierarchical design enables sustained selenium delivery, effectively targeting prostate cancer *via* AGGF1/Wnt/β-catenin suppression and oxidative stress mitigation. Mechanistically, we identified AGGF1 as a novel orchestrator of prostate tumorigenesis through redox-Wnt crosstalk. The dual-layer architecture enhances cellular uptake and sustains therapeutic selenium levels (> 72 h), addressing rapid clearance and toxicity of prior systems. These findings redefine AGGF1 beyond angiogenesis, highlighting its role as a redox-metabolic master regulator in prostate cancer and validating Se@SiO_2_@mSiO_2_ as a precision nanotherapeutic platform.

Previous studies have found that AGGF1 plays a crucial role in the development of prostate cancer,^[18, 19, 20, 21, 22, 23, 24, 25]^ but its functional role in prostate cancer remained unexplored. Here, through comprehensive bioinformatics analysis and clinical specimen validation, we show that the high expression of AGGF1 may indicate a higher degree of tumor malignancy, a later stage and a poorer prognosis in patients with prostate cancer. Mechanistically, AGGF1 was associated with Wnt/ β-catenin pathway, which can inhibits the expression of β-catenin and the phosphorylation of GSK3β, and reduces oxidative damage at the same time, playing a role in dual-pronged inhibiting tumor growth. This discovery has expanded the theoretical model of “oxidative stress-WNT pathway interaction” proposed by Zhang *et al*.^[26]^: AGGF1 may act as a molecular sensor of oxidative stress signal, regulate the transcriptional activity of Wnt pathway by regulating the level of oxidative stress, and form a signal loop. At the same time, this study supplemented the observation of Sochacka *et al*.^[27]^ at the mechanism level that selenium compounds can inhibit oxidative stress by regulating glutathione peroxidase (GPx). This is also similar to our previous research results that nano-selenium can inhibit cellular oxidative stress. At the same time, we confirmed through *in vivo* and *in vitro* experiments that Se@SiO_2_@mSiO_2_ can inhibit the proliferation of prostate cancer cells by down-regulating AGGF1, and its effect is double dependent on concentration and time. It is worth noting that compared with the mechanism reported by Song *et al*.^[28]^ that monolayer Se@SiO_2_ regulates oxidative stress through the PI3K/AKT pathway, the regulatory network of the AGGF1-WNT pathway found in this study is more specific- overexpression of AGGF1 can reverse the anti-proliferation effect of nanomaterials. However, knockdown of β-catenin had no significant effect on the expression of AGGF1, which further supported the positioning of AGGF1 as the upstream signal hub of the Wnt pathway. Western Blot results showed that Se@SiO_2_@mSiO_2_ could simultaneously inhibit the expression of β-catenin and phosphorylation of GSK3B, and could also effectively reduce the binding ability of AGGF1 to β-catenin, which was consistent with the “AGGF1-Wnt/β-catenin cascade” model proposed by Yao *et al*.^[10]^ in gastric cancer. The nuclear translocation and accumulation of β-catenin are the markers of Wnt pathway activation, driving the transcription of a large number of proliferative and anti-apoptotic genes including c-Myc and Cyclin D1. The significant reduction in its expression level strongly suggests that Se@SiO_2_@mSiO_2_ effectively inhibits the activity of this pathway in prostate cancer cells. While during the treatment with Se@SiO_2_@mSiO_2_, overexpression of AGGF1 through transfection technology can partially reverse the downregulation of β-catenin protein expression caused by Se@SiO_2_@mSiO_2_. This further proves that the inhibitory effect of Se@SiO_2_@mSiO_2_ on the Wnt/β-catenin pathway is functionally dependent on its negative regulation of AGGF1 protein level. AGGF1 is located at the upstream of this regulatory cascade reaction, and its expression level change directly controls the abundance and activity of downstream β-catenin. Therefore, we believe that AGGF1 may act as a “molecular switch” that can be precisely regulated by Se@SiO_2_@mSiO_2_ nanomaterials, controlling the initiation of the carcinogenic signal flow. Meanwhile, in this study, we found that Se@SiO_2_@mSiO_2_ regulates both Wnt pathway and oxidative stress by targeting AGGF1, which provides a theoretical basis for the future development of dual-pathway inhibitors.

Aiming at the problems such as low drug loading of Se@SiO_2_ and sudden release of selenium ions developed in our previous research, Se@SiO_2_@mSiO_2_ was synthesized by double-layer coating technology in this study, which significantly improved the stability and slow release performance of the material. Transmission electron microscopy and XRD analysis confirmed that Se@SiO_2_@mSiO_2_ has a uniform mesoporous structure and good crystal integrity. Compared with the previously developed mesoporous silica drug delivery system, the cumulative release period of selenium ions is extended to more than 72 h, which meets the long-term controlled release requirement of tumor therapy. In addition, FITC labeling experiments showed that the nanoparticles showed uniform cytoplasmic distribution in cancer cells. Histopathological and serological evaluation further verified the *in vivo* safety of Se@SiO_2_@mSiO_2_, which had significantly lower hepatorenal toxicity than traditional selenium preparations (such as sodium selenite) ,^[29]^ due to the precise regulation of selenium ion release kinetics by the double-layer coating system. Furthermore, our research also revealed that the toxicity of Se@SiO_2_@mSiO_2_ towards prostate cancer cells was significantly lower than that of non-malignant prostate epithelial cells. We speculate that this might be because the inhibitory effect of Se@SiO_2_@mSiO_2_ on the proliferation of tumor cells is mainly achieved by suppressing the intracellular oxidative stress level (This has been well demonstrated in Figure 8). However, non-malignant prostate epithelial cells have stronger antioxidant reserves, which can effectively buffer ROS impacts, resulting in the fact that Se@SiO_2_@mSiO_2_’s further reduction of ROS on these cells is not significant. Additionally, from the physical and chemical properties of the silica shell, the weakly acidic environment of lysosomes within cancer cells makes it easier for the SiO_2_ shell layer to dissolve and for selenium to be released. We believe that these could be the reasons why Se@SiO_2_@mSiO_2_ exerts a significantly stronger antiproliferative effect on PCa cells compared to non-malignant prostate epithelial cells.

Meanwhile, many previous studies have confirmed that bilayer spherical shell nanomaterials have other diverse structural advantages. For instance, the research of Wang *et al*.^[30]^ found that compared with single-shell nanomaterials, multi-shell nanomaterials, due to their structure where multiple shells are independent of each other and nested layer by layer, can control the composition of their components through the interfaces between different shells. Even each interface can carry different types of drugs to exert different functions, thereby precisely controlling the chemical reaction environment. This is also in line with our material structure design concept. In this study, Se ions inside of the double-layer silica spherical shell nanomaterials can be released along the concentration gradient based on the different concentrations in different layers, thereby achieving the purpose of sustained release and controlled release. Some studies also suggest that due to the large specific surface area of multi-shell nanomaterials, they have higher application efficiency. Although such multi-shell nanomaterials have been studied less in the medical field, they have been relatively mature in the field of physics. For example, Dong ^[31]^ *et al*. constructed five-shell SnO_2_ microspheres and found that they could significantly enhance the light conversion efficiency. Therefore, the application of double-layer SiO_2_ spherical shells in this study also lays a foundation for the subsequent cross-disciplinary field of medicine and materials science, especially the application of double-layer nanomaterials. On the other hand, in this study, Se was loaded into nanomaterials to exert the effect of tumor-killing. This is because Se is a key ion in the biological metabolic process, and our previous studies have also confirmed that local low concentrations of Se can inhibit the level of oxidative stress, thereby accelerating the repair of tissue damage. However, local high concentrations of Se can induce an increase in oxidative stress levels, thereby regulating the occurrence and development of tumors.^[11, 12, 13, 32, 33]^ However, due to the small therapeutic dose and toxic dose windows of Se, cytotoxicity is prone to occur during the treatment process, thus limiting its clinical application. Therefore, by constructing a nanocarrier material that can carry a sufficient amount of Se and can be sustainably released at a stable rate over a long period of time, we can achieve the therapeutic effect on tumors while reducing its side effects. Meanwhile, due to the excellent biocompatibility and material plasticity of nanomaterials, they can be designed into various dosage forms and clinical application methods such as injection, oral administration, topical application, and topical application, further enhancing the convenience and wide range of their application.

Although this study systematically elucidated the molecular mechanism of Se@SiO_2_@mSiO_2_ inhibiting prostate cancer through AGGF1, there were still the following limitations: (1) The sample size was relatively limited, and patients with metastatic castration-resistant prostate cancer (mCRPC) were not included. In the future, the cohort should be expanded to verify the prognostic value of AGGF1; (2) The upstream molecular mechanism of AGGF1 regulating Wnt pathway has not been fully clarified, and whether AGGF1 plays a role through tyrosine kinase receptors (such as VEGFR) or epigenetic modifications (such as DNA methylation) needs to be further explored; (3) The subcutaneous transplanted tumor model used in animal experiments failed to fully simulate the microenvironment characteristics of prostate cancer bone metastasis, and orthotopic transplantation or organoid models could be used to improve the efficacy evaluation. (4) At present, the synthesis efficiency of Se@SiO_2_@mSiO_2_ is not particularly stable, and the synthesis process can be further improved in the future to increase its efficiency. (5) Furthermore, based on the results of previous studies and our own observations during the experiment, it was found that the therapeutic window of Se ions is extremely narrow. Even a very low concentration of exogenous Se ions can lead to cell death. This makes it extremely difficult to simply add Se ions as a control group in our experiment. This is the main reason why we did not set up this control group. In the future, if the concentration of added Se ions can be more precisely regulated, and its pharmacological effects can be exerted while ensuring cell survival, we will further improve the related experiments.

Future research can further improve and enhance the targeting and controllability of nanomaterials. For example, by loading prostate-specific membrane antigen (PSMA) antibodies onto Se@SiO_2_@mSiO_2_ to further enhance its tumor-specific enrichment ability. At the same time, the combined therapeutic potential of Se@SiO_2_@mSiO_2_ can be further explored by combining it with Wnt/β-catenin pathway inhibitors or antioxidants. Relevant cell and animal experiments can be conducted to verify whether this can further enhance the therapeutic effect of prostate cancer. In terms of the exploration of molecular mechanisms, future research can further investigate the transcriptional activation mechanism of AGGF1 as an upstream regulatory molecule in the ROS and Wnt/β-catenin pathways.^[34]^ For instance, using Methyl-Seq to examine the epigenetic modifications in the promoter region of AGGF1, or Co-IP assay to further screen out transcription factors that can directly bind to AGGF1, and verify their interaction networks. In this study, it was found that AGGF1 might be a key hub in the process where Se@SiO_2_@mSiO_2_ acts on prostate cancer cells and triggers intracellular signal cascades. Further exploration of its direct target is an important depth in the mechanism study. Future research can utilize methods such as molecular docking, *in vitro* protein binding experiments, and mass spectrometry analysis to deeply investigate whether and how Se@SiO_2_@mSiO_2_ interacts with the AGGF1 protein. Secondly, in addition to the discovered ROS and Wnt/ β-catenin pathways, other targets of AGGF1 can also be further revealed by means of multi-omics integrated analysis.^[35]^ In terms of sample size, although the clinical data of the 54 patients included in this study can provide the evidence related to the association between AGGF1 and the malignancy and prognosis of prostate cancer, future studies will increase the sample size to further verify the prognostic value of AGGF1. Furthermore, in the future, by improving patient-derived organoids (PDO) or patient-derived xenografts (PDX) models, it will be possible to more realistically simulate the *in vivo* microenvironment of tumors, thereby evaluating the pharmacological effects of new therapies and making the results closer to clinical practice.

## Conclusion

In this study, a new double-layer silica nano-selenium material Se@SiO_2_@mSiO_2_ was constructed, which revealed the regulatory role of AGGF1 as a key factor of Wnt/β-catenin pathway and oxidative stress in prostate cancer for the first time, and verified the therapeutic potential of nanomaterials through multi-dimensional experiments. The results provide theoretical support for the development of precision therapy strategies based on AGGF1 targeting, and open up a new path for the design of multifunctional nanocarriers.
